# Calculated parenteral initial treatment of bacterial infections: Microbiology

**DOI:** 10.3205/id000062

**Published:** 2020-03-26

**Authors:** Michael Kresken, Béatrice Grabein, Karsten Becker, Eberhard Straube, Thomas A. Wichelhaus, Birgit Willinger

**Affiliations:** 1 Antiinfectives Intelligence GmbH, Campus Hochschule Bonn-Rhein-Sieg, Rheinbach, Germany; 2 Rheinische Fachhochschule Köln gGmbH, Cologne, Germany; 3 Stabsstelle Klinische Mikrobiologie und Krankenhaushygiene, Klinikum der Universität München, Munich, Germany; 4 Institut für Medizinische Mikrobiologie, Universitätsklinikum Münster, Germany; 5 Institut für Medizinische Mikrobiologie, Universitätsklinikum Jena, Germany; 6 Institut für Medizinische Mikrobiologie und Krankenhaushygiene, Universitätsklinikum Frankfurt, Germany; 7 Klinisches Institut für Labormedizin, Medizinische Universität Wien, Vienna, Austria

## Abstract

This is the second chapter of the guideline “Calculated initial parenteral treatment of bacterial infections in adults – update 2018” in the 2^nd^ updated version. The German guideline by the Paul-Ehrlich-Gesellschaft für Chemotherapie e.V. (PEG) has been translated to address an international audience.

Preliminary microbiological findings regarding the patient and their immediate environment are crucial for the calculation of treatment with antibiotics in each case, as well as the resistance situation of the ward on which the patient is being cared for. If such data is not available, regional or supra-regional data can be used as a fallback. This chapter describes the methods of susceptibility testing, informs about the resistance situation in Germany and describes the main resistance mechanisms of bacterial pathogens against antibiotics. Further, the chapter informs about collateral damage of antibiotics as well as medical measures against increasing resistance.

## Zusammenfassung

Dies ist das zweite Kapitel der von der Paul-Ehrlich-Gesellschaft für Chemotherapie e.V. (PEG) herausgegebenen S2k Leitlinie „Kalkulierte parenterale Initialtherapie bakterieller Erkrankungen bei Erwachsenen – Update 2018“ in der 2. aktualisierten Fassung.

Entscheidend für die Kalkulation einer Therapie mit Antibiotika im Einzelfall sind vorausgehende mikrobiologische Befunde des Patienten selbst und seiner unmittelbaren Umgebung sowie die Resistenzsituation der Abteilung, in der der Patient versorgt wird. Sind solche Daten nicht verfügbar, kann auf regionale oder überregionale Daten zurückgegriffen werden. Dieses Kapitel beschreibt die Methoden der Empfindlichkeitsprüfung, informiert über die überregionale Resistenzsituation in Deutschland und beschreibt die wichtigsten Resistenzmechanismen bakterieller Krankheitserreger gegen Antibiotika. Ferner informiert das Kapitel über Kollateralschäden von Antibiotika sowie medizinische Maßnahmen gegen die zunehmende Resistenz.

## Introduction

The rational use of antibiotics, including the consideration of economic aspects, can only be done on the basis of well-founded microbiological data collected directly from the patient or their immediate environment. This includes knowledge of the pathogen spectrum of an infection (e.g. pneumonia, cholecystitis, urinary tract infection); the results of screening tests for the detection of multidrug-resistant bacteria in relation to in-patient admission; the history of previous stays in other medical facilities and stays abroad; knowledge of the constantly changing, local or regional but also national and global resistance situation. 

In addition, this knowledge should be incorporated into hospital hygiene management. Here, close cooperation between the treating physician the microbiologists and hygiene doctors respectively is essential. Cooperation begins with pre-analytics, i.e. the selection and correct removal as well as the best possible transfer of the relevant material for examination of the suspected or existing infection, since errors that occur here cannot be corrected. In addition, information on the infection and on the hospital or travel history is necessary for the examiner, since this information may be used as appropriate to indicate specific methods for detecting (multidrug-resistant) infectious agents.

Despite considerable progress in molecular biology, cultivation of the pathogens remains a mandatory requirement for adequate susceptibility testing. DNA-based molecular tests can only detect selected resistance genes of bacteria or fungi but cannot provide any information on the resistance phenotype. For a pathogen culture it is necessary to obtain a sufficient amount of high-quality test material (tissue samples and aspirates are better than smears!). Cooperation between the clinic and the microbiology lab is continued by a joint specialist evaluation of the micro-organisms detected and their antibiotic susceptibility for a clinical diagnosis as well as by agreement on the rational antibiotic treatment and, if necessary, initiation of hospital hygiene measures. Close coordination between clinical and medical microbiology/hospital hygiene should culminate in joint development and enforcement of local guidelines on the use of antibiotics (“antibiotic stewardship”), pathogen surveillance and hygienic anti-epidemial measures. Of particular importance here is that the clinical microbiologist/hospital hygienist is available on-site to regularly attend ward rounds as a form of infectiology consultation and ad-hoc case discussions. This allows a targeted diagnosis, avoids unnecessary effort and ensures a rational antibiotic therapy.

## Susceptibility testing

The susceptibility of a pathogen to an antibiotic is determined by in vitro activity. The reference method is the determination of the minimum inhibitory concentration (MIC in mg/l) according to ISO 20776-1 [1]. In the laboratory routine mostly derived methods are used, which should fulfill ISO 20776-2 [2]. In addition, the agar diffusion test is also used. The specific instructions of the Microbiological-Infectiological Quality Standards (MiQ) of the German Society for Hygiene and Microbiology (DGHM) as well as the principles of quality assurance according to the guidelines of the German Medical Association for the quality assurance of laboratory medical examinations (Rili-BÄK) must be followed [3].

The numerical value of the MIC and the inhibition diameter (in mm) gives information about the susceptibility of a pathogen in vitro. To create a microbiological profile, a species-specific interpretation of the antibiogram is usually required. The clinical interpretation of the result is done using limit concentrations (thresholds) in the categories *susceptible* (S), *intermediate* (I, if defined) or *resistant* (R). For most antibiotics, European harmonized limits have now been established by the European Committee of Antimicrobial Susceptibility Testing (EUCAST) (http://www.eucast.org/clinical_breakpoints/). EUCAST had called for the establishment of National Antibiotic Susceptibility Test Committees in order to establish the EUCAST breakpoints in European laboratories and, if necessary, adapt them to national circumstances. On the initiative of representatives of the German Society for Hygiene and Microbiology (DGHM), the Paul Ehrlich Society for Chemotherapy (PEG) and the Robert Koch-Institute (RKI), a National Antibiotic Susceptibility Test Committee (NAK) of the EUCAST was founded in Germany (http://www.nak-deutschland.org). In Austria, the National Antimicrobial Susceptibility Testing Committee Austria (NAC-AT; https://www.analyse.eu/content/inhalte/nationales_referenzzentrum/nac_at/) has taken on this task.

The breakpoints set by EUCAST and NAK take into account the doses authorized in Germany; they are included in the technical information and therefore are part of the marketing approval for the medicinal product concerned. For this reason, the breakpoints of the US Clinical Laboratory Standards Institute (CLSI) should no longer be taken into account. The determination of the pathogen susceptibility by means of MIC determination offers the advantage over the agar diffusion test that it not only provides a qualitative (S, I, R), but also a quantitative test result. Knowledge of the MIC is especially important if therapeutic drug monitoring is carried out to check adequate drug concentrations.

In cases of doubt and in the case of therapy-critical resistance results, with established pathogen identity, additional methods of nucleic acid detection (e.g. PCR) or antigen detection (e.g. PBP2a detection) may underpin the evaluation of specific susceptibilities for selected pathogens. The interpretation aids used in automatic resistance determination methods do not replace the specialist evaluation of the examination result on a case-by-case basis.

Even optimal microbiological diagnosis cannot rule out a discrepancy between the antibiogram and the clinical outcome of treatment. The most common causes are errors in the pre-analytical phase, which lead to the investigation not of the causative agent but of another bacterial strain. A drop in quality also occurs during extended transport time of test samples, which can easily lead to a shift in the microbiological flora such as death of susceptible pathogens, overgrowth of isolated pathogens and dehydration of the material. The reasons for clinical failure in susceptible pathogens or clinical success in resistant pathogens may be diverse in nature and are summarized in Table 1 (Tab. 1). All in all, it has to be said that susceptibility testing (antibiogram) based on current standards has technical limitations – depending on the method – and does not always correlate with the clinical situation but helps to estimate the clinical effectiveness of an antibiotic! Furthermore, susceptibility testing provides the necessary data on pathogen epidemiology on-site as a basis for a locally-adapted, calculated antibiotic therapy.

## Resistance situation

Preliminary microbiological findings regarding the patient and their immediate environment are crucial for the calculation of treatment with antibiotics in each case, as well as the resistance situation of the ward on which the patient is being cared for. If such data is not available, regional or supra-regional data can be used as a fallback. The supra-regional resistance situation in clinically important bacterial species in the hospital area is examined at regular intervals by the working party *Antimicrobial Resistance* of the PEG in selected laboratories in Germany, Austria and Switzerland using uniform and standardized methods (PEG resistance study, https://www.p-e-g.org/resistenzdaten.html) Original data is processed as measured MIC values. Current data on the resistance situation are also provided by other initiatives that process partially interpreted resistance data from different systems, such as the Antibiotics Resistance Surveillance (ARS) of the Robert Koch-Institute (RKI, https://ars.rki.de/) and the National Reference Center (NRZ) for Surveillance of Nosocomial Infections with the KISS projects (http://www.nrz-hygiene.de/surveillance/kiss/) and SARI (http://sari.eu-burden.info/). The European Antimicrobial Resistance Surveillance Network (EARS-Net), coordinated by the European Center for Disease Prevention and Control (ECDC), provides country-specific national resistance data for isolates of patients with systemic infections (https://ecdc.europa.eu/en/about-us/partnerships-and-networks/disease-and-laboratory-networks/ears-net). Further data sources for the monitoring of the most common infectious agents in hospitals are provided by (inter-)national resistance surveillance studies of the pharmaceutical industry, regional networks (e.g. antibiotic resistance monitoring in Lower Saxony ARMIN (http://www.nlga.niedersachsen.de/infektionsschutz/armin_resistenzentwicklung/antibiotika-resistenz-monitoring-in-niedersachsen-armin-19418.html), as well as various other NRZ (https://www.rki.de/DE/Content/Infekt/NRZ/nrz_uebersicht_gesamt_node.html). A summary of data on antimicrobial use and the spread of antibiotics resistance in human and veterinary medicine can be found in the report GERMAP (https://www.p-e-g.org/germap-27.html), which goes back to a joint initiative of the Federal Office for Consumer Protection and Food Safety (BVL), PEG and the Department of Infectiology in Freiburg and is updated regularly.

Since 1975, the PEG resistance study has been conducted using qualified laboratories. Subproject H (Hospital) of the study carried out in 2013 examined 5,852 bacterial pathogen isolates from various sample materials (wound material 29%, airway material 23%, blood 12%, urinary tract material 11%, other 26%) in 25 laboratories. Approximately 64% of the samples were from general care wards, 26% from ICU patients and 10% from outpatients. The following section presents the most important results of this study as well as some data from ARS on the resistance situation in blood culture isolates in 2015 [4]. The results of the working party* Antimicrobial Resistance *of the PEG originate mainly from laboratories at maximum care hospitals. They can therefore not be readily transferred to the situation in other care areas. 

Multidrug resistant pathogens can pose significant difficulties in antibiotic treatment. In many cases the frequency of resistance and resistance patterns of pathogens of nosocomial infections correlate with the selection and frequency of antibiotics used in the hospital concerned. A calculated antibiotic therapy must take account of the pathogen epidemiology and the intra-station resistance situation. In intensive care units in particular, regular collection of these data is an indispensable prerequisite for successful treatment. Overall, however, in the clinical area, absolute usage figures are likely to play a lesser role than non-compliance with general hygiene measures and infection control measures to prevent pathogen transmission.

### Beta-lactam antibiotics

According to the 2013 PEG resistance study, resistance to ampicillin was 50.8% for *Escherichia coli* (n=596) and 18.3% for cefuroxime. The proportion of isolates with the extended spectrum beta-lactamase (ESBL) phenotype, which can also inactivate cephalosporins of groups 3–5 (as per the classification of the cephalosporins, see [5]), was 15.4% in *Escherichia coli* and 17.8% in *Klebsiella pneumoniae* (n=304). The proportion of cefotaxim-resistant blood culture isolates was 11.5% for *Escherichia coli* (n=9,958) and 13.0% for *Klebsiella pneumoniae* (n=1,796). Enterobacteriaceae (in particular *Klebsiella pneumoniae*) with resistance to group 1 carbapenems (imipenem, meropenem) are also already endemic in Germany. However, their prevalence is (still) below 1%.

Of the *Pseudomonas aeruginosa* isolates of the resistance study (n=733), 13.4% showed resistance to ceftazidime and 19.4% resistance to piperacillin/tazobactam. Blood culture isolates were 9.1% resistant to ceftazidime (n=1,076) and 15,6% resistant to piperacillin/tazobactam (n=1,073). The proportion of strains with intermediate susceptibility or resistance to imipenem and meropenem was approximately 15–17% for the isolates of patients in general care wards and 25–30% for the isolates of intensive care patients, both in the resistance study and in the blood culture isolates.

The resistance rates of *Acinetobacter baumannii* isolates to imipenem and meropenem in the resistance study (n=88) were 28.4% and 29.5%, respectively. No *Acinetobacter pittii* isolates (n=85) with resistance to imipenem or meropenem were found.

The proportion of methicillin (cefoxitin/oxacillin)-resistant strains in *Staphylococcus aureus* isolates (MRSA) has trended downwards in recent years; it was 13.5% in the resistance study (n=748) and 11.8% in the blood culture isolates (n=7,740). In contrast, the rate of methicillin (oxacillin)-resistant isolates in *Staphylococcus epidermidis* (n=466) was approximately 75% and in *Staphylococcus haemolyticus* (n=95) >90%. In the case of ARS, no species-related information can be found on the resistance situation of coagulase-negative staphylococci. Overall, 58.8% of blood culture isolates of coagulase-negative staphylococci (n=27,804) showed resistance to oxacillin.

The proportion of strains resistant to ampicillin in *Enterococcus faecium* was 90.6% in the resistance study isolates (n=320) and 93.3% in the blood culture isolates (n=1,270). In contrast, 100% of the *Enterococcus faecalis* isolates of the resistance study (n=424) and >99% of the blood culture isolates (n=1,705) were ampicillin-susceptible.

Penicillin-resistant pneumococci (MIC >2 mg/l) are still (very) rare in Germany. In the resistance study, no resistant strain was found among the clinical isolates (n=432), while 2% of the blood culture isolates (n=980) were rated as penicillin-resistant. The rate of isolates with intermediate penicillin susceptibility (MIC 0.25–2 mg/l) was 10.6% in the resistance study and 4.3% in the blood culture isolates.

### Fluoroquinolones

The proportion of ciprofloxacin-resistant strains in the resistance study was 24.7% for *Escherichia coli*, 16.8% for *Klebsiella pneumoniae* and 16.6% for *Pseudomonas aeruginosa*. The resistance rates for levofloxacin were 24.3% (*Escherichia coli*), 12.2% (*Klebsiella pneumoniae*) and 20.9% (*Pseudomonas aeruginosa*). The *Staphylococcus aureus* isolates of the resistance study showed 19.4% resistance to moxifloxacin. Of the blood culture isolates, 20.7% (*Escherichia coli*, n=11,611), 12.1% (*Klebsiella pneumoniae*, n=2,051) and 13.8% (*Pseudomonas aeruginosa*, n=1,076) were resistant to ciprofloxacin and 20.8% (*Staphylococcus aureus*, n=5,369) to moxifloxacin.

### Macrolides

The rate of macrolide-resistant pneumococci (test substance erythromycin) was 11.8% in the isolates of the resistance study (n=432) and 7.9% in the blood culture isolates (n=944).

### Glycopeptides

The resistance situation regarding *Staphylococcus aureus* remains favorable. While vancomycin-resistant MRSA strains (VRSA, MIC >8 mg/l) based on the *vanA* resistance mechanism are extremely rare worldwide, in many countries so-called MRSA-VISA (vancomycin-intermediate *Staphylococcus aureus* with an MIC of 4–8 mg/l according to the criteria of CLSI; vancomycin-resistant according to the criteria of EUCAST) are observed, with changes in the cell wall considered to be responsible for the decreased susceptibility amongst other things. As possible precursors in the development towards VISA, there are increasing numbers of isolates that appear to be vancomycin-susceptible in testing but often contain subpopulations of organisms with elevated MIC values (≥4 mg/l) (heterogeneous VISA, hVISA) [6], [7], [8]. In addition, in some studies a gradual, average increase in vancomycin MIC for MRSA and MSSA below the respective limits has been reported (referred to in the literature as “MIC creep” or “MIC shift”) [9], [10], [11], [12]. Other studies were not able to prove this effect [13], [14]. An increased MIC of vancomycin, however, is of general importance since it has been shown that the bactericidal activity of a fixed concentration of vancomycin on MRSA is already reduced at a MIC of 2 mg/l and that a high failure rate of vancomycin therapy is associated with bacteremic infections by such agents [15], [16], [17]. In the 2013 PEG resistance study, no glycopeptide-resistant *Staphylococcus aureus* isolate was found. The highest MIC was 2 mg/l for vancomycin and 1 mg/l for teicoplanin. Likewise, no vancomycin-resistant isolate was found among the coagulase-negative staphylococci tested in the resistance study. However, 35.8% of the *Staphylococcus epidermidis* isolates and 37.9% of the *Staphylococcus haemolyticus* isolates were teicoplanin resistant.

The proportion of vancomycin-resistant strains in the *Enterococcus faecium* isolates reached 16.6% in the 2013 resistance study. Of these, 7.5% showed the VanA phenotype (resistant to vancomycin and teicoplanin) and 9.1% the VanB phenotype (resistant to vancomycin and susceptible to teicoplanin). In contrast, only one vancomycin-resistant isolate (VanB phenotype) was found in *Enterococcus faecalis*. Of the *Enterococcus faecium* blood culture isolates (n=1,729), 12.2% were vancomycin-resistant, while the blood culture isolates of *Enterococcus faecalis* (n=2,288) were 99.9% vancomycin-susceptible. In case of infection by strains with the VanB phenotype, the use of teicoplanin may lead to development of resistance [18].

### Trimethoprim/sulfamethoxazole

In the resistance study, 29.0% of the *Escherichia coli* isolates were resistant and 26.4% of the blood culture isolates (n=11,605).

### Daptomycin, linezolid, tigecycline, colistin, fosfomycin

The resistance situation of daptomycin and linezolid in staphylococci (including MRSA), enterococci (including VRE) and streptococci is (still) very favorable worldwide. Development of resistance during treatment is nonetheless possible, as with all antibiotics [19], [20], [21], [22]. However, a plasmid-encoded resistance mechanism against oxazolidinone has been described in staphylococci [23], [24] and enterococci [25], [26] which may favor the spread of resistant strains. 

Tigecycline-resistant Gram-positive pathogens are also (still) very rare at present. Isolates of *Escherichia coli* (including ESBL-producing strains) are almost always tigecycline-susceptible, while 5–10% of the isolates of *Enterobacter cloacae* and *Klebsiella pneumoniae* are considered resistant [27]. In *Acinetobacter baumannii* and *Klebsiella pneumoniae*, development of resistance is possible during treatment [28], [29], [30]. Imipenem-resistant strains of *Acinetobacter baumannii* are more likely to show decreased susceptibility to tigecycline than imipenem-susceptible strains [31].

Colistin is a potential alternative to treat infections caused by multidrug-resistant Gram-negative pathogens. Representatives of Proteeae such as *Proteus* spp. and *Serratia* spp. are naturally colistin-resistant. The resistance study found a single colistin-resistant *Escherichia coli* isolate. The transmissible gene *mcr-1* was found to be the responsible resistance gene [32]. The isolates of *Enterobacter aerogenes* (n=60), *Enterobacter cloacae* (n=197) and *Klebsiella pneumoniae* showed 3–5% resistance to colistin. In contrast, all of the isolates tested for *Pseudomonas aeruginosa* and *Acinetobacter baumannii* were colistin-susceptible.

The proportion of Enterobacteriaceae isolates with fosfomycin resistance varied considerably from species to species and in the resistance study for *Escherichia coli* it was 1.8%, for *Klebsiella pneumoniae* 20.1% and for *Enterobacter cloacae* 35.5%.

Further evidence-based information on the resistance situation of important bacterial pathogens can be found in Table 2 (Tab. 2).

## Mechanisms of resistance to antibiotics

The classical resistance mechanisms of bacteria essentially fall into three groups:

- Antibiotic-inactivating enzymes
- Altered or missing target structures
- Altered access to target structures (increased efflux, reduced influx)

The resistance-encoding genetic determinants may be intrinsic to the bacterial chromosome; however, they are often found on mobile genetic elements on and/or off the chromosomes (e.g. resistance plasmids, transposons, insertion sequences, genomic islands, and antibiotic resistance cassettes) which are responsible for the rapid horizontal spread of resistance among bacteria.

In addition, there are phenotype-related mechanisms of resistance, which can lead to a lack of susceptibility or limited susceptibility to antibiotics which in vitro tested as susceptible [33], [34], [35]. These include, amongst other things, the formation of biofilms on natural or abiotic surfaces (e.g. foreign body associated infections), the invasion of pathogens into host cells and/or the expression of the small colony phenotype or similar forms (dormant forms, persisters) with a change in metabolism which impacts the effects of antibiotics. In part, the use of an antibiotic itself may lead to the formation of such phenotypes.

## Collateral damage of antibiotics

Collateral damage refers to undesirable environmental effects of antibiotic use, such as the displacement of the normal flora in favor of hospital bacteria or fungi, selection of antibiotic-resistant microorganisms in the normal flora, the occurrence of *Clostridium difficile*-associated diarrhea and the colonization and infection with multidrug-resistant pathogens. At the top of the list of multidrug-resistant pathogens are Enterobacteriaceae, *Pseudomonas aeruginosa* and *Acinetobacter baumannii* with 3MRGN/4MRGN status [36] and MRSA and vancomycin-resistant *Enterococcus faecium* (VRE). Epidemiological studies have shown the risk of collateral damage for various antibiotics.

Patients with Gram-negative bacterial infections treated with fluoroquinolones are at an increased risk of infections caused by fluoroquinolone-resistant pathogens [37]. This relationship was shown in a study, amongst others, of patients with urinary tract infections with a significantly increased risk of ciprofloxacin-resistant *Escherichia coli* in patients who had been treated with ciprofloxacin more than once in the year prior to the urinary tract infection [38]. Another study found a significant correlation between the frequency of fluoroquinolone resistance in *Escherichia coli* in patients with community-acquired urinary tract infections and the level of fluoroquinolone consumption in the population [39]. Moreover, there is evidence that the use of fluoroquinolones also increases the risk of acquiring MRSA and ESBL-producing pathogens [37], [40]. The relationship can be explained by the fact that the majority of MRSA and ESBL-producing strains show resistance to fluoroquinolones.

Several case-control studies have also described group 3 cephalosporins as a risk factor for ESBL-producing pathogens. They have also been identified as a risk factor for MRSA and VRE infections and are also likely to be a risk for the acquisition of carbapenemase-producing pathogens, as the latter may also inactivate cephalosporins [37].

Carbapenems are highly important in the treatment of life-threatening infections. As a result of the increase in ESBL-producing pathogens, which can no longer be treated with cephalosporins and usually no longer with fluoroquinolones, the importance of carbapenems has increased significantly. Since it is unlikely that antibiotics with new mechanisms of action against Gram-negative bacteria will be approved in the coming years, an increase in carbapenem resistance would have dramatic consequences for treatment. It has already been shown that the use of imipenem and meropenem is associated with a higher risk of colonization by MRSA, ciprofloxacin-resistant *Pseudomonas aeruginosa* and VRE than the use of cephalosporins, fluoroquinolones or piperacillin/tazobactam [41]. Carbapenems are also a risk factor for infections with *Stenotrophomonas maltophilia*.

## Medical measures against increasing resistance

The development of resistance in bacteria during medical treatment is based on genetic variability and selection of rarely occurring resistant variants through the use of antibiotics. The main goals for mitigating resistance must be to lower the selection pressure and prevent the transmission of (multi) resistant pathogens. The following measures can influence the development of resistance and the spread of resistant bacteria:

- Well-founded, targeted use of antibiotics aimed at the individual patient
- Adequate dosage and duration of treatment
- Combination treatment (in the same dosage as the individual substances) with a high probability of treatment failure in the presence of primarily resistant pathogens, e.g. empirical treatment of severe infections such as pneumonia or sepsis with suspected involvement of *Pseudomonas aeruginosa*
- Parallel use of different antibiotic classes for the same indication
- Adaption of treatment once plausible microbiological findings are to hand
- Strict indication of treatment for the prophylactic and topical use of antibiotics
- Strict adherence to hygienic hand disinfection as well as further measures for prevention of infection
- Continuous compilation of pathogen and resistance statistics (local, regional to [supra]national) as a basis for hospital hygiene measures and guidelines for antibiotic therapy (§23 Abs.1 IfSG)
- Monthly report to clinicians on patients populated and infected with (multi) resistant pathogens, with assessment of epidemiological development and derivation of specific hygiene measures [36]
- Continuous, prospective recording of nosocomial infections in defined (possibly rolling) clinical areas, with assessment and derivation of hygiene measures (§23 IfSG)
- Continuous surveillance regarding the occurrence of *Clostridium difficile* (patient-related, Robert Koch-Institute [42])
- Screening (detection swab) of newly admitted patients for (multi) resistant pathogens, e.g. MRSA and 4MRGN according to current guidelines of the Hospital Hygiene Commission [36], [43]
- Ongoing, continuous screening for defined pathogens in neonatology as specified by KRINKO [44]
- Continuous professional education in the field of antibiotic treatment and prevention and control of multidrug-resistant pathogens
- Ensuring rational hospital antibiotic use through the establishment of Antibiotic Stewardship (ABS) expert teams, consisting of at least one specialist for infectious diseases (or a clinically active specialist with training in infectious diseases), a microbiology, virology and infection epidemiology specialist for microbiological diagnostics and clinical microbiological advice and the local physician responsible for hospital hygiene as well as an experienced specialist pharmacist for clinical pharmacy/hospital pharmacy [45]
- Interdisciplinary cooperation of all occupational groups involved in the treatment of infections (specialist for infectious diseases or a clinically active specialist with training in infectious diseases; microbiology, virology and infection epidemiology specialist for microbiological diagnostics and a local physician responsible for hospital hygiene) through joint infectiology case conferences
- Vaccinations

## Note

This is the second chapter of the guideline “Calculated initial parenteral treatment of bacterial infections in adults – update 2018” in the 2^nd^ updated version. The German guideline by the Paul-Ehrlich-Gesellschaft für Chemotherapie e.V. (PEG) has been translated to address an international audience.

## Competing interests

The authors declare that they have no competing interests.

## Figures and Tables

**Table 1:**
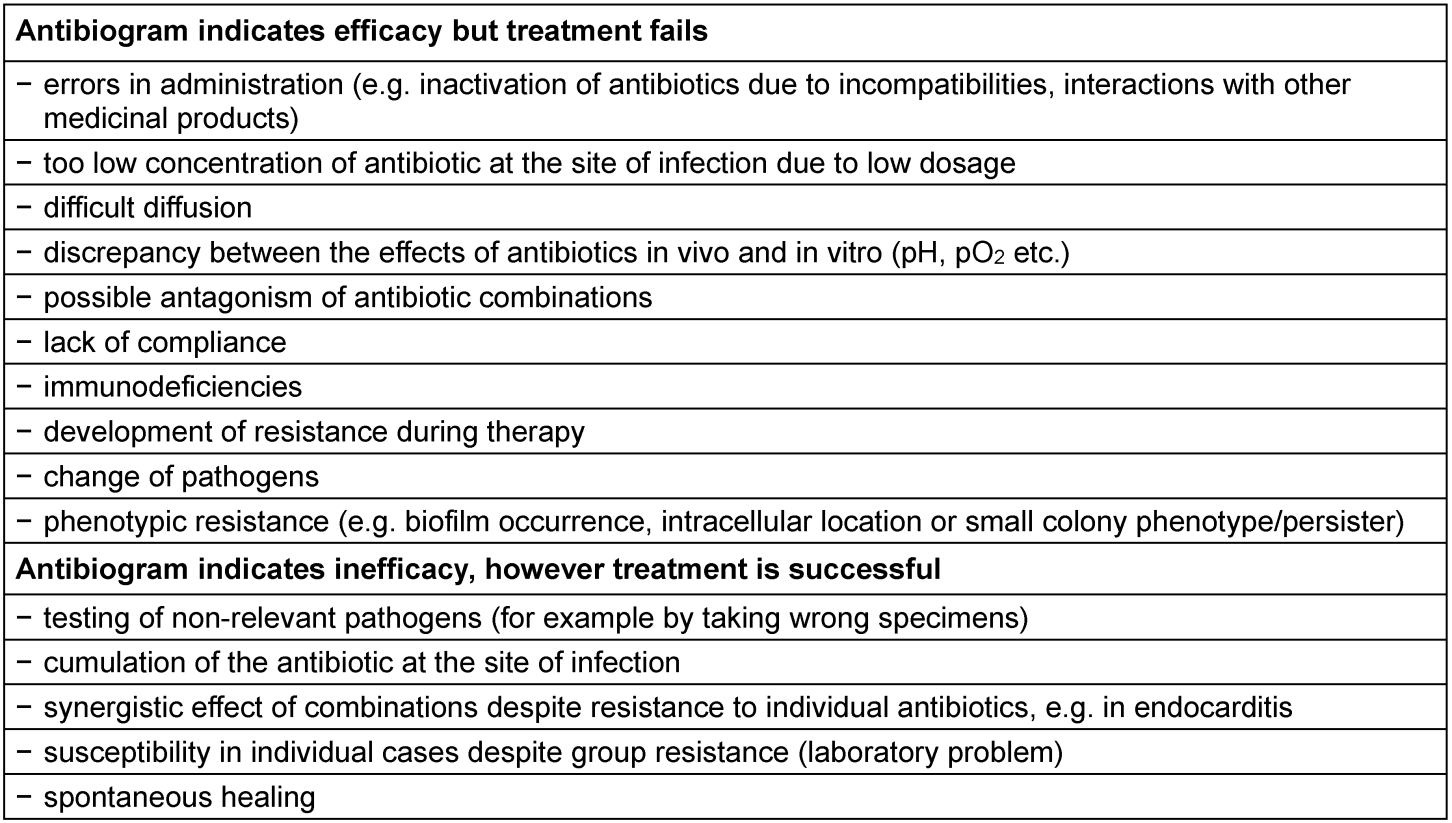
Reasons for discrepancies between antibiogram and outcome of clinical therapy

**Table 2:**
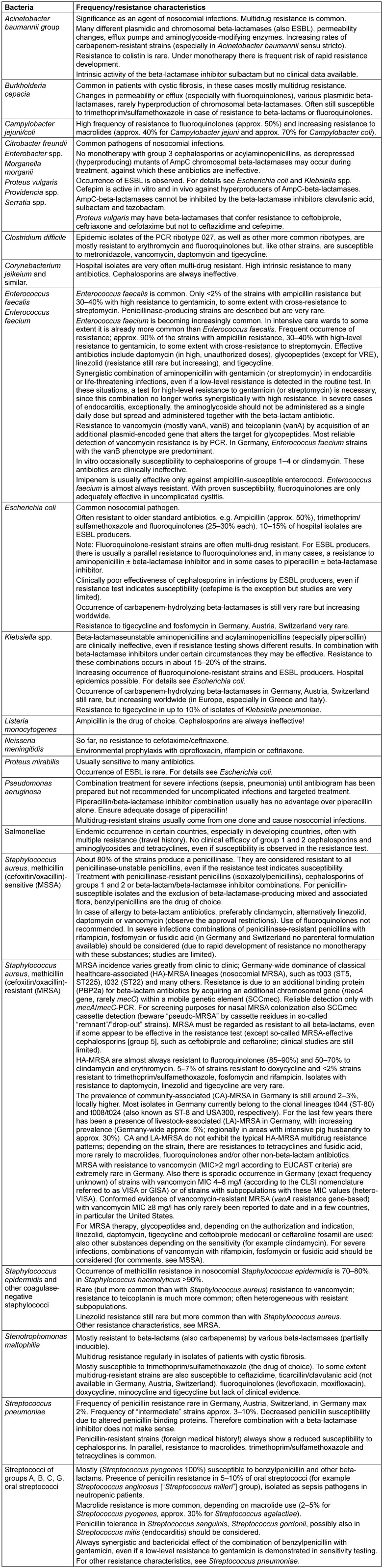
Information on the resistance situation in important bacterial pathogens
